# Adjuvant therapy for retroperitoneal sarcoma: a meta-analysis

**DOI:** 10.1186/s13014-021-01774-w

**Published:** 2021-10-07

**Authors:** Xiangji Li, Tong Wu, Mengmeng Xiao, Shanshan Wu, Li Min, Chenghua Luo

**Affiliations:** 1 Department of Retroperitoneal Tumor Surgery, Peking University International Hospital, 1ShengMingYuan Road, Beijing, 102206 People’s Republic of China; 2 Department of Sports Medicine, Peking University Third Hospital, Institute of Sports Medicine of Peking University, Beijing Key Laboratory of Sports Injuries, Beijing, People’s Republic of China; 3 Clinical Epidemiology and EBM Unit, National Clinical Research Center for Digestive Disease, Beijing Friendship Hospital, Capital Medical University, Beijing, People’s Republic of China; 4 Department of Gastroenterology, National Clinical Research Center for Digestive Disease, Beijing Digestive Disease Center, Beijing Key Laboratory for Precancerous Lesion of Digestive Disease, Beijing Friendship Hospital, Capital Medical University, Beijing, People’s Republic of China

**Keywords:** Meta-analysis, Retroperitoneal sarcoma, Adjuvant therapy, Surgery

## Abstract

**Background:**

Adjuvant therapy is a promising treatment to improve the prognosis of cancer patients, however, the evidence base driving recommendations for adjuvant radiotherapy (ART) or chemotherapy (ACT) in retroperitoneal sarcomas (RPS) primarily hinges on observational data. The aim of this study was to evaluate the effectiveness of adjuvant therapy in the management of RPS patients.

**Methods:**

We searched PubMed, Web of Science, Embase, ASCO Abstracts, and Cochrane Library for comparative studies (until December 2020) of adjuvant therapy versus surgery alone. Data on the following endpoints were evaluated: overall survival (OS), local recurrence (LR), recurrence-free survival (RFS), and metastasis-free survival (MFS). Data were summarized as hazard ratios (HR) with 95% confidence intervals (CI). Risk of bias of studies was assessed with Begg’s and Egger’s tests.

**Results:**

A total of 15 trials were eligible, including 9281 adjuvant therapy and 21,583 surgery alone cases (20 studies for OS, six studies for RFS, two studies for LR, and two studies for MFS). Meta-analysis showed that ART was associated with distinct advantages as compared to surgery alone, including a longer OS (HR = 0.80, *P* < 0.0001), a longer RFS (HR = 0.61, *P* = 0.0002), and a lower LR (HR = 0.31, *P* = 0.005). However, this meta-analysis failed to demonstrate a benefit of ACT for RPS patients, including OS (HR = 1.11, *P* = 0.19), RFS (HR = 1.30, *P* = 0.09) and MFS (HR = 0.69, *P* = 0.09). In the sensitivity analysis, ACT was associated with a worse OS (HR = 1.19, *P* = 0.0002). No evidence of publication bias was observed.

**Conclusions:**

Overall, the quality of the evidence was moderate for most outcomes. The evidence supports that ART achieved a generally better outcome as compared to surgery alone.

**Supplementary information:**

The online version contains supplementary material available at 10.1186/s13014-021-01774-w.

## Introduction

Retroperitoneal sarcomas (RPS) are the second common malignancy after soft tissue sarcoma (STS) of the extremities, accounting for 10–15% of all STS and 30% of all malignant retroperitoneal tumors [1]. R0 surgical resection is the most potential treatment to cure patients with localized disease, which means the adjacent organs invaded are often not preserved. According to current studies, the rate of complete resection ranges from 41.8 to 76% [2]. However, local recurrence remains high even if negative margins is being achieved as far as possible, and leads to poor prognosis, with 5-year overall survival (OS) ranging from 39 to 65% and a mortality rate of 20–75% [3–7].

To improve local control and overall survival, adjuvant therapy (AT) such as adjuvant radiotherapy (ART) has been investigated. However, there are insufficient evidences to compile treatment guidelines due to the different conclusions based on limited retrospective clinical studies (RCSs) [8–22]. For example, multiple analyses of the Surveillance, Epidemiology, and End Results (SEER) database and a retrospective analysis from French have shown that ART could not improve OS in RPS patients [5, 9, 12, 23], but Trovik et al. [8] and others [10, 18] demonstrated a significant improvement in OS as well as recurrence-free survival (RFS) in patients undergoing ART, which are obviously confusing. In addition, most trials were undertaken in the setting of advanced extremity sarcomas and the generalizability of these data is limited [24, 25], resulting chemotherapy cannot be as a standard approach to treat RPS. Therefore, it is greatly clinical significance to discuss whether advanced RPS patients can benefit from adjuvant chemotherapy (ACT).

The aim of this meta-analysis is to review the latest body of literature comparing AT with surgery alone in RPS patients, and to clarify the role of ART and ACT in the prognostic outcome of these patients.

## Methods

### Searching strategy

The meta-analysis was conducted according to the guidelines of the Cochrane Handbook, and reported according to the Preferred Reporting Items for Systematic Reviews and Meta-Analyses (PRISMA) statement. We search PubMed, Web of Science, Embase, ASCO Abstracts, and Cochrane library for eligible studies between January 2000 and December 2020 with the searching strategy: (((adjuvant radiotherapy OR postoperative radiotherapy OR postoperative chemotherapy) AND (retroperitoneal sarcomas OR retroperitoneal soft tissue sarcomas OR retroperitoneal neoplasms)) AND (surgery))). In addition, reference lists of all studies were screened to identify potentially eligible studies.

### Selection criteria

Studies were included based on following criteria: 1) randomized clinical trial (RCT), case–control study, or retrospective cohort study of AT versus surgery for RPS patients; 2) PRS confirmed by pathological biopsy; 3) studies providing data of hazard ration (HR) and 95% confidence interval (CI) of local recurrence (LR), metastasis-free survival (MFS), RFS or OS of AT versus surgery for RPS patients. The exclusion criteria included: (1) letter, editorial or noncomparative study; 2) the cases or the groups in the study were less than 20 and 5 respectively; (3) HR and 95% CI cannot be extracted from studies; 4) non-human studies.

### Data extraction and quality assessment

Two authors independently extracted data using a standard form from eligible studies. Discrepancies were resolved by consensus, and invited a third investigator to interpret if the differences remained controversial after discussion. The following information was extracted from each included study: primary author, year of publication, patient source, study type, number of patients, age, high malignancy grade, tumour size, intervention, surgical margins, dose of radiotherapy (drug of chemotherapy), and data of OS, LR, RFS and MFS. All included studies were assessed by Newcastle–Ottawa Scale (NOS). The assessment tool focused on three aspects includes participant selection, comparability and exposure with 9 items. A study was considered of high quality if it scored 7 points or higher.

### Statistical analysis

Meta-analysis was performed by Review Manager version 5.4 (Cochrane Collaboration, London, UK). HR was used as a summary statistic and calculated using either fixed-effects models or, in the presence of heterogeneity (*P* < 0.10, *I*^2^ > 50%), random-effects models. Sensitivity analysis was used to identify the possible sources of heterogeneity and detect the stability of studies by re-meta-analysis with one involved study excluded each time. Publication bias was assessed using Begg’s and Egger’s test with stataCorp version 15.1 (College Station, TX 77,845, USA). All P-values were two-sides.

## Results

### Search results and characteristic of included studies

**Fig. 1:**
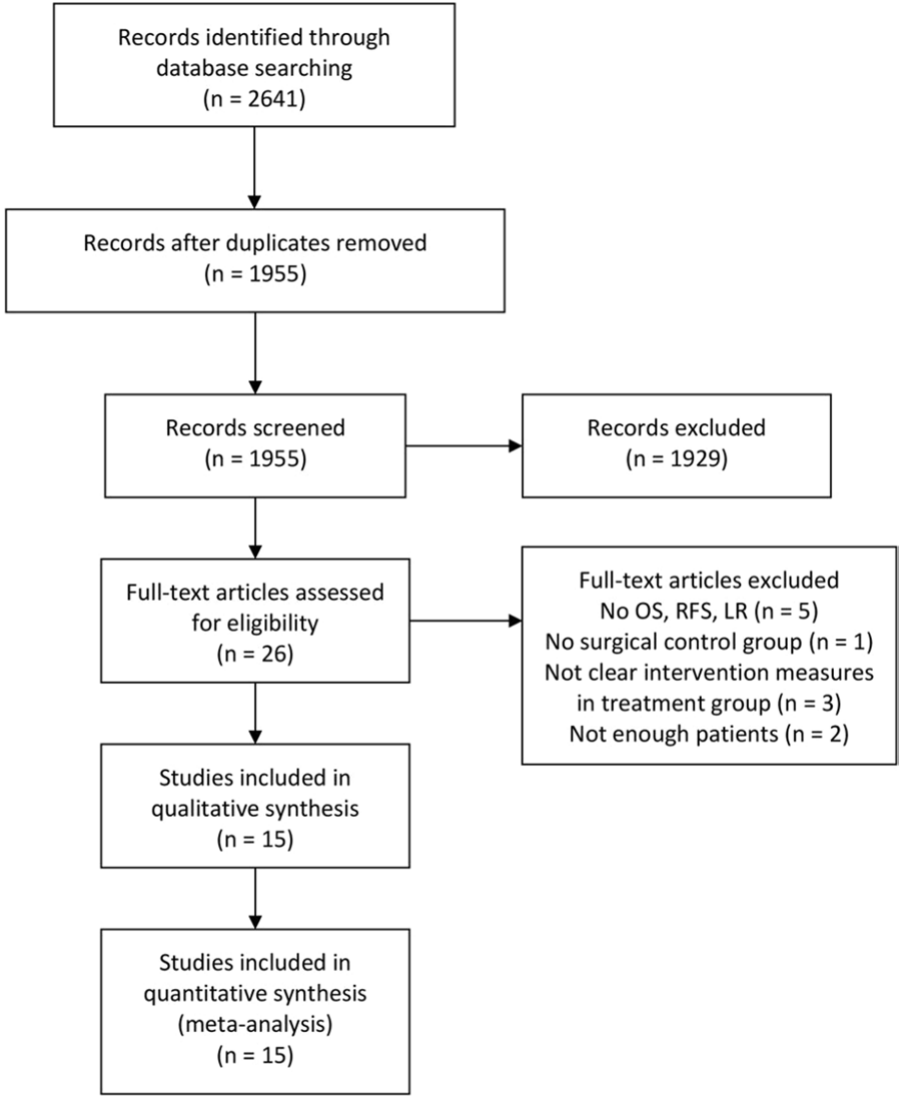
Preferred reporting items for systematic reviews and meta-analyses (PRISMA). Flowchart of studies included in the review with reasons for exclusion

The detailed characteristics of included studies and the results of the quality assessment are summarized in Table 1. A total of 2641 references were identified form databases of PubMed, Web of Science, Embase, ASCO Abstracts and Cochrane library. After selection according to the inclusion/exclusion criteria, 15 RCSs were eligible for meta-analysis (Fig. 1). In these studies, 30,864 patients with RPS were compared, including 9281 patients who underwent adjuvant therapy and 21,583 patients who underwent surgery alone. In addition, five studies performed concurrent ART versus surgery and ACT versus surgery, four studies divide into adjuvant therapy group and surgery group with propensity score marched (PSM). The earliest study was published in 2008, and the latest in 2019. Studies were conducted in four different countries (USA, France, Norway and Italy). All studies were evaluated by NOS and the overall quality averaged 7.45 stars (range 7–8) on scale of 0–9 (Additional file 1: Table S1).

**Table 1:** Characteristics of included studies

Study (author, year)	Patient source	Type of study	Number of patients (treat/control)	Age (years)		High malignancy grade		Tumour size (cm)		Intervention (treat/control)	Surgical margins		Radiotherapy/chemotherapy	Outcome	HR (95%CI)	NOS score
				Treat	Control	Treat (%)	Control (%)	Treat	Control		Treat (Neg/Pos)	Control (Neg/Pos)	Dose (Gy)/ Drug			
Adjuvant radiotherapy																
Nussbaum et al. 2016^a (19)^	USA	RCS	2196/2196	59.5(13.9) ^#^	59.4(15.3) ^#^	63	62	12.8(12) ^#^	12.9(11.5)	Sur + RT/Sur	1338/858	1377/819	50(45–54) ^¶^	OS	0.78 (0.71–0.85)	8
Trovik et al. 2014^(8)^	Norway	RCS	42/55	61(35–82) ^§^	63(15–83) ^§^	34	37	19(6–60) ^§^	19(4–43) ^§^	Sur + RT/Sur	25/17	29/26	< 50	OS	0.36 (0.18–0.72)	8
														MFS	0.42 (0.20–0.88)	
														LR	0.20 (0.09–0.45)	
Bates et al. 2018^(10)^	USA	RCS	144/336	–	*–*	–	–	–	–	Sur + RT/Sur	–	–	–	OS	0.42 (0.19–0.90)	7
Zhou et al. 2010^(13)^	USA	RCS	364/1183	–	–	–	–	–	–	Sur + RT/Sur	–	–	–	OS	0.78 (0.63–0.95)	7
Lepechoux et al. 2013^(12)^	France	RCS	56/42	50(21–80) ^§^	54(19–77) ^§^	40	24	15(6–46) ^§^	20(4–60) ^§^	Sur + RT/Sur	–	–	50.4(14–62) ^§^	OS	0.91 (0.34–1.39)	8
														RFS	0.43 (0.20–0.88)	
Chouliaras et al. 2019^a (21)^	USA	RCS	59/59	58.2(52–70) ^¶^	65.8(52–77) ^¶^	74.6	71.2	12.5(8.5–20) ^¶^	15(8–23) ^¶^	Sur + RT/Sur	34/25	32/27	50.4(45, 54.3) ^¶^	OS	0.80 (0.47–1.35)	8
														RFS	0.70 (0.43–1.14)	
														LR	0.47 (0.21–1.06)	
Miura et al. 2015^a† (18)^	USA	RCS	938/3050	–	–	–	–	$\le 10$(26.8%)	$\le 10$(27.7%)	Sur + RT/Sur	–	–	–	OS	0.79 (0.70–0.90)	8
								10–20(34.5%)	10–20(35.0%)							
								$\ge 20$(17.7%)	$\ge 20$(18.8%)							
Gronchi et al. 2012^† (17)^	Italy	RCS	101/230	57(48–67) ^¶^		35		17(10–26) ^¶^		Sur + RT/Sur	Neg/Pos: 305/26		50(36–65) ^§^	OS	0.64 (0.41–1.00)	7
														RFS	0.57 (0.35–0.92)	
														MFS	2.38 (1.34–4.42)	
Gronchi et al. 2009^† (16)^	Italy	RCS	88/200	55(47–66) ^¶^		35.8		16(10–26) ^¶^		Sur + RT/Sur	Neg/Pos: 257/31		50 (36–65) ^§^	OS	0.55 (0.35–0.86)	7
														RFS	0.65 (0.42–1.01)	
														MFS	1.47 (0.81–2.68)	
Stahl et al. 2017^† (15)^	USA	RCS	1132/2772	60^§^		52.2		16(0.3–90) ^§^		Sur + RT/Sur	Neg/Pos: 2593/1422		50.4(45–54.1) ^¶^	OS	0.81 (0.70–0.93)	8
Tseng et al. 2011^(9)^	USA	RCS	373/1130	61.5(14.8) ^#^		31.8		15.5(0.5–99.5) ^§^		Sur + RT/Sur	Neg/Pos: 660/875		–	OS	0.92 (0.78–1.09)	7
Klooster et al. 2016^† (20)^	USA	RCS	102/293	63(53–72) ^¶^		49		20(12–44) ^¶^		Sur + RT/Sur	Neg/Pos: 0/395		–	OS	0.78 (0.58–1.05)	7
Berger et al. 2018^(22)^	USA	RCS	550/2212	62.9(11.2) ^#^		39.1		19.9(11.9) ^#^		Sur + RT/Sur	Neg/Pos: 1445/1317		–	OS	0.80 (0.68–0.94)	7
Nathan et al. 2009^(5)^	USA	RCS	254/1365	63^§^		37		17(0.5–99) ^§^		Sur + (RT + IORT)/Sur	–		–	OS	0.95 (0.78–1.15)	7
Adjuvant chemotherapy																
Datta et al. 2017^(11)^	USA	RCS	390/377	$\le 62$(72.3%) 63–71(20.0%) $\ge 72$(7.7%)	$\le 62$(67.1%) 63–71(24.1%) $\ge 72$(8.8%)	77.2	78	$\le 10$(24.4%) 10–20(48.5%) $\ge 20$(27.1%)	$\le 10$(23.3%) 10–20(47.6%) $\ge 20$(29.1%)	Sur + CT/Sur	180/210	170/207	–	OS	1.30 (1.05–1.61)	8
Miura et al. 2015^a‡ (18)^	USA	RCS	1525/1525	55(43–64)	57(48–64)	30.2	29.8	$\le 10$(26.8%) 10–20(34.5%) $\ge 20$(17.7%)	$\le 10$(27.7%) 10–20(35.0%) $\ge 20$(18.8%)	Sur + CT/Sur	614/911	629/896	–	OS	1.17 (1.04–1.31)	8
Klooster et al. 2016^‡ (20)^	USA	RCS	122/273	63(53–72) ^¶^		49		20(12–44) ^¶^		Sur + CT/Sur	Neg/Pos: 0/395		–	OS	1.09 (0.83–1.45)	8
Gronchi et al. 2009^‡ (16)^	Italy	RCS	182/394	55(47–66) ^¶^		35.8		16(10–26) ^¶^		Sur + CT/Sur	Neg/Pos: 257/31		–	OS	1.30 (0.86–1.97)	7
														RFS	1.34 (0.88–2.05)	
														MFS	0.72 (0.39–1.24)	
Gronchi et al. 2012^‡ (17)^	Italy	RCS	218/444	57(48–67) ^¶^		35		17(10–26) ^¶^		Sur + CT/Sur	Neg/Pos: 305/26		–	OS	1.14 (0.74–1.75)	7
														RFS	1.26 (0.80–1.97)	
														MFS	0.66 (0.36–1.20)	
Stahl et al. 2017^‡ (15)^	USA	RCS	445/3447	60^§^		52.2		16(0.3–90) ^§^		Sur + CT/Sur	Neg/Pos: 2593/1422		–	OS	0.82 (0.67–0.99)	8

CI, confidence interval; DSS, disease-specific survival; HR, hazard ratio; LR, local recurrence; Neg, negative; OS, overall survival; Pos, positive; RCS, retrospective cohort study; RCT, randomized clinical trial; RFS, recurrence-free survival; RT, radiotherapy, Sur, Surgery

^a^Propensity score matched (PSM); ^†,‡^Adjuvant radiotherapy (†) and adjuvant chemotherapy (‡) in the same study; ^#^ Data are mean (SD); ^§^ Data are median or median (range); ^¶^ Data are median (IQR)

### Meta-analysis of OS

**Fig. 2:**
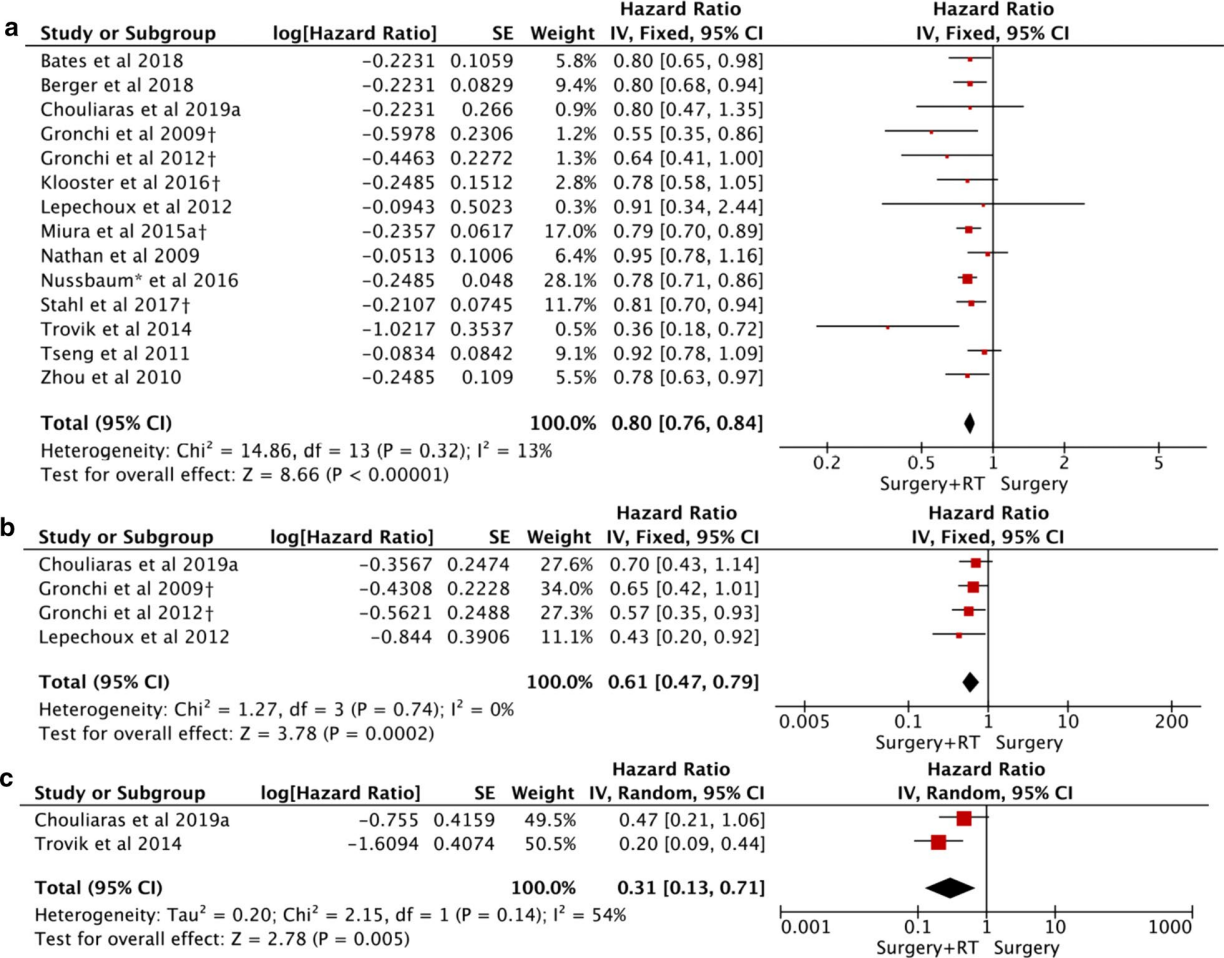
Forest plot and pooled analysis of hazard ratio for overall survival (OS) (**a**), recurrence-free survival (RFS) (**b**), and local recurrence (LR) (**c**) in RPS patients with adjuvant radiotherapy

**Fig. 3:**
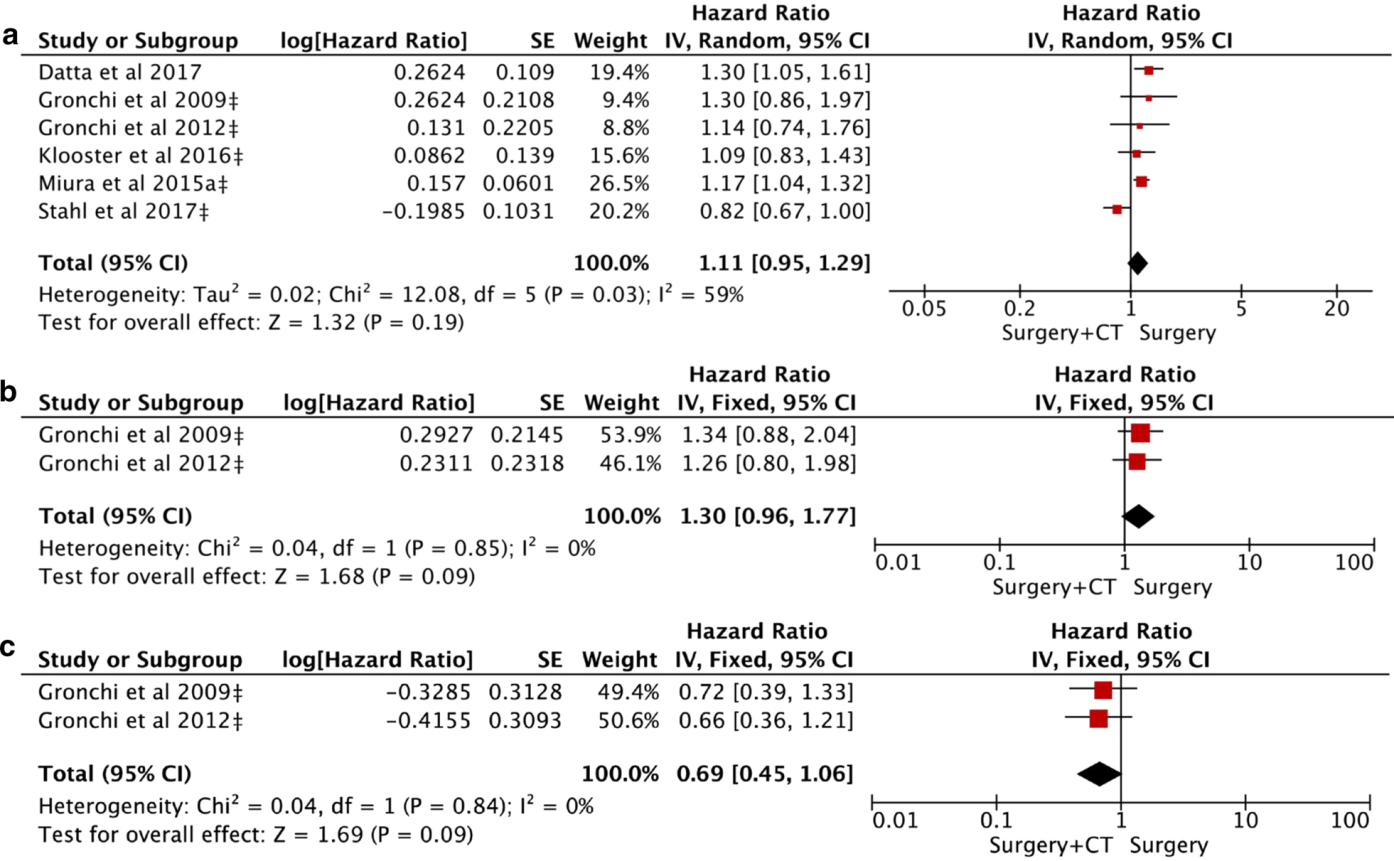
Forest plot and pooled analysis of hazard ratio for overall survival (OS) (**a**), recurrence-free survival (RFS) (**b**), and metastasis-free survival (MFS) (**c**) in RPS patients with adjuvant chemotherapy

The fixed-effects results showed that the OS was significantly improved in ART group as compared to surgery alone group (HR = 0.80, 95% CI 0.76–0.84; *P* < 0.0001) (Fig. 2a). However, there was no significant difference in ACT group versus surgery alone group (HR = 1.11, 95% CI 0.95–1.29; *P* = 0.19) (Fig. 3a). Notable heterogeneity was seen in the latter, and the sensitivity analysis indicated that patients benefited more from surgery alone than ACT (HR = 1.19, 95% CI 1.08–1.30; *P* = 0.0002).

### Meta-analysis of RFS

The meta-analysis in the fixed-effects model indicated that RFS was obviously improved in ART group (HR = 0.61, 95% CI: 0.47–0.79; *P* = 0.0002), and the four sets of results showed no significant amount of heterogeneity (Fig. 2b). However, there was no significant difference in ACT versus surgery (HR = 1.30, 95% CI 0.96–1.77; *P* = 0.09), no statistical heterogeneity was found (Fig. 3b).

### Meta-analysis of LR

LR was reported in two PSM studies, including 101 patients in ART group and 114 patients in surgery group. The result showed LR of ART group was much lower than surgery group (HR = 0.31, 95% CI 0.13–0.71; *P* = 0.005) (Fig. 2c).

### Meta-analysis of MFS

The MFS was reported by two studies in ACT versus surgery, 1238 participants. There was no statistical significance between the two comparisons (HR = 0.69, 95% CI 0.45–1.06; *P* = 0.09), no statistical heterogeneity was found (Fig. 3c).

### Publication bias

**Fig. 4:**
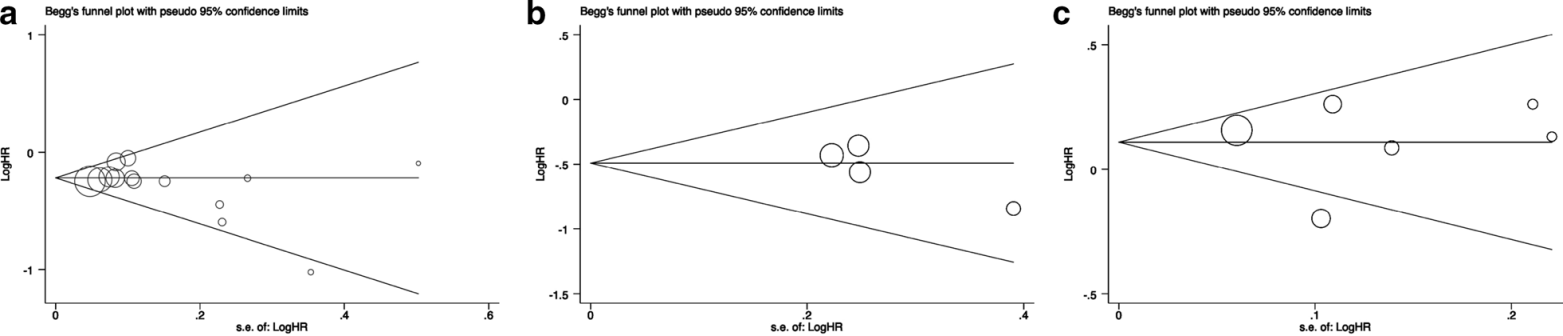
Begg’s test for overall survival (OS) and recurrence-free survival (RFS) in RPS patients with adjuvant radiotherapy (**a**,** b**), and overall survival (OS) in RPS patients with adjuvant chemotherapy (**c**)

The detailed results of pooled analysis and the heterogeneity analysis are summarized in Table 2. Publication bias was determined by Begg’s and Egger’s tests, and there was no evidence of publication bias for OS and RFS (Fig. 4a–c and Additional file 1: Table S2). However, there were significant heterogeneity in two pooled analysis, sensitivity analysis showed that one of which had a different result after excluding the most heterogeneous study. We will analyze this in the later part of discussion.

**Table 2:** Summary of results

Categories	Studies	Patients	Model	HR (95%CI)			Heterogeneity		
				value	z	P-value	X^2^	I^2^	P-value
OS									
Adjuvant radiotherapy	14	20,564	Fixed	0.80 (0.76–0.84)	8.66	< 0.0001	14.86	13%	0.32
Adjuvant chemotherapy	6	9342	Random	1.11 (0.95–1.29)	1.32	0.19	12.08	59%	0.03
Sensitivity analysis of adjuvant chemotherapy	5	5450	Fixed	1.19 (1.08–1.30)	3.68	0.0002	1.35	0%	0.85
RFS									
Adjuvant radiotherapy	4	1454	Fixed	0.61 (0.47–0.79)	3.78	0.0002	1.27	0%	0.74
Adjuvant chemotherapy	2	1238	Fixed	1.30 (0.96–1.77)	1.68	0.09	0.04	0%	0.85
LR									
Adjuvant radiotherapy	2	215	Random	0.31 (0.13–0.71)	2.78	0.005	2.78	54%	0.14
MFS									
Adjuvant chemotherapy	2	1238	Fixed	0.69 (0.45–1.06)	1.69	0.09	0.04	0%	0.84

CI, confidence interval; HR, hazard ratio; LR, local recurrence; OS, overall survival; RFS, recurrence-free survival

## Discussion

The unique biological behavior of RPS brings great challenges to clinicians in the treatment of this disease. R0 resection is the only potentially curative therapy, but the risk of LR is substantial because large size and anatomical structure of the tumor frequently preclude resection with widely clear margins. Five-year LR rates range from 28 to 60% [16, 26–28]. With long-term follow-up, almost all patients are likely to recur [29]. Therefore, it is urgent to clarify the comprehensive treatment of RPS. ART, remains a controversial component of treatment, unlike STS in extremity, there is no rigorous level I data to support ART significantly reduce LR in RPS patients, and lessons from ART trials in extremity STS are difficult to translate directly to the RPS due to the potential for significant toxicity at equivalent dose [26]. Here our pooled analysis revealed that LR of RPS patients is distinctly reduce in ART group as compared to surgery alone, it is a key parameter to evaluate the prognosis of patients under different treatments. Besides, whether ART is beneficial to OS is also controversial. Several RCSs indicated consistently that ART improve local control (LC) and RFS but failed to demonstrate a statistically significant association with OS [5, 9, 12, 21]. Others obtained opposite results that ART not only improved the OS, but also has obvious curative effect in RFS and LC with a premise that the margin of surgical resection reaches R0 or R1 [3, 10, 11, 13, 16, 18]. In our meta-analysis, the results support the latter.

[In addition to therapeutic effects, radiation toxicities are primary consideration in selecting an appropriate radiation dose. Among the included studies, detailed radiation doses were given in seven studies (Table 1), and the median radiation doses were all 50 Gy, with the highest not exceeding 65 Gy. However, radiotherapy toxicities and target volumes were not mentioned in any of these studies, and the main reason was the lack of records on the aspect in the databases selected by these retrospective studies. Besides, the location occupied by the original sarcomas would be filled with adjacent normal tissues after resection, which also led to uncertainty in the radiotherapy target volumes. Previous two trials (phase1 and phase 2) have been reported that 45-54 Gy were optimal doses according to the potential benefits and risks assessed by them, to avoid bowel complications reported with higher doses and the potential negative impact on surgery [30, 31]. Recently, a phase 3 trials (multicenter, open-label, randomized) analysed the radiation toxicities in the ART, radiotherapy was delivered as 50.4 Gy (in 28 daily fractions of 1.8 Gy). The resulted showed the most common grade 3–4 adverse events (lymphopenia, anaemia, and hypoalbuminaemia) were reported in 127/127 patients in ART group, and in 16/128 patients in surgery alone. Serious adverse events were reported in 30/127 patients in ART group, and in 13/128 patients in surgery alone. One patient was died in ART group due to gastropleural fistula [32]. Complications rates were lower in this trial than have been reported with ART, ranging from20-40% in retrospective series [12, 33, 34]. In our study, the radiation doses performed were basically consistent with the optimal doses Therefore, we considered that the radiation toxicities are acceptable, but the specific radiation toxicities need to be further investigated.]

Anthracyclines were the first systemic chemotherapeutic agents to demonstrate activity in STS, and doxorubicin was main representative. According currently studies, using ACT in STS could not benefit patients, neither patients whose tumors remained resectable or patients who had metastasized at an advanced stage. A multicenter phase III RCT (EORTC) [35] randomly assigned 351 patients with non-metastatic macroscopically resected II-III tumors to the postoperative chemotherapy group (175 patients with ifosfamide/doxorubicin) or to the control group (176 patients). The results demonstrated OS did not differ significantly between groups (HR = 0.94, 95% CI 0.68–1.31; *P* = 0.72) nor did RFS (HR = 0.91, 95% CI 0.67–1.22; *P* = 0.51). A retrospective study of efficiency of ACT in resected RPS [11], published in 2017, showed that utilization of ACT was associated with worse long-term survival (HR = 1.30, 95% CI 1.05–1.61; *P* = 0.017). However, the trend of OS improvement with ACT were found in spindle cell (HR = 0.37, 95% CI 0.10–1.38), giant cell (HR = 0.82, 95% CI 0.32–2.13) and synovial sarcoma (HR = 0.26, 95% CI 0.05–1.33). Here our findings show that ACT cannot benefit RPS patients from OS, RFS and MFS, and even as previously reported study, it may cause worse OS (sensitivity analysis). Although whether metastasis occurs for patients is not the main reason to cause the increase in mortality, MFS was also included in the study as a prognostic parameter for PRS. In the studies we reviewed, only two studies included MFS [16, 17], and the two studies were conducted by the same author in different years.

In our study, notable heterogeneity was seen in LR of ART and OS of ACT, and only OS can perform sensitivity analysis due to lack of studies in LR. The sensitivity analysis showed that ACT was associated with worse OS, which was different from previous results of pooled analysis. In any case, the two results support that ACT cannot improve OS in patients with RPS. From another perspective, we have to admit that the result is not robust. Meanwhile, some limitations should be considered before appraising the results of this study. First, interventions in the treatment group in some studies were not limited to postoperative radiotherapy and chemotherapy, and in order to minimize the interference factors, we extracted HR form the multivariate COX regression analysis. Second, all included studies were RCSs, no RCT was found in databases we search. Finally, studies were insufficient in the sub-analysis of LR of ART and RFS of ACT duo to only two studies were included, limiting the validity of the comparisons between studies and conclusions drawn.

In this study, the quality of evidence was moderate but sufficient to establish the efficacy of ART for RPS. The relationship between ACT and the prognosis of RPS needs to be further studied, especially for patients with resectable RPS. Since there was a trend that ART is more likely to improve OS and LC of RPS patients, while ACT is for MFS, distinguishing the different efficiency between ART and ACT was also urgent. In addition, further studies could significantly change the results that ACT was associated with a wore OS, and more prognostic factors, such as pathological type, surgical resection method, dose, and related toxic complications, need to be included for analysis. Of course, all results will eventually need to be verified by multicentered RCTs.

## Conclusions

Overall, our Meta-analysis showed RPS patients who underwent ART had better prognostic outcome than those who underwent surgery alone, including a longer OS, a longer RFS, and a lower LR. However, Those positive therapeutic effects have not been demonstrated in ACT, either in OS, RFS or MFS.

## Supplementary Information

**Additional file 1.** The Quality of Enrolled Studies, and The Publication Bias of Subgroup Analysis.** Table S1**. The Quality of Enrolled Studies.** Table S2**. Begg’s and Egger’s Test for Publication Bias.

## Data Availability

The datasets used and/or analyzed during the current study are available from the corresponding author on reasonable request.

## Abbreviations

ART: adjuvant radiotherapy
ACT: adjuvant chemotherapy
RPS: retroperitoneal sarcomas
OS: overall survival
LR: local recurrence
RFS: recurrence-free survival
MFS: metastasis-free survival
HR: hazard ratios
CI: confidence intervals
AT: adjuvant therapy
STS: soft tissue sarcoma
RCS: retrospective clinical study
SEER: surveillance, epidemiology, and end results
PRISMA: preferred reporting items for systematic reviews and meta-analyses
RCT: randomized clinical trial
NOS: Newcastle–Ottawa scale (NOS)
PSM: propensity score marched
LC: local control
