# Supplementary material for: Adjuvant therapy for retroperitoneal sarcoma: a meta-analysis
Source: Radiat Oncol. 2021 Oct 7;16:196. doi: 10.1186/s13014-021-01774-w (PMC8496039; doi:10.1186/s13014-021-01774-w)
Supplement: Supplementary file 1 — Additional file 1. The Quality of Enrolled Studies, and The Publication Bias of Subgroup Analysis. Table S1. The Quality of Enrolled Studies. Table S2. Begg’s and Egger’s Test for Publication Bias. [file 13014_2021_1774_MOESM1_ESM.docx]

**Appendix 1: Supplemental Tables**

**Table S1. The Quality of Enrolled Studies**

| **Newcastle Ottawa Score** | | | | | | | | | | |
| --- | --- | --- | --- | --- | --- | --- | --- | --- | --- | --- |
| **Study (author, year)** | **Case selection** | | | | **Comparability** | | **Outcome assessment** | **Follow-up** | **Sufficiency** | **Overall score** |
|  | **1** | **2** | **3** | **4** | **1** | **2** |  |  |  |  |
| Nussbaum et al 2016^a (19)^ | * | * | * |  | * | * | * | * | * | 8 |
| Trovik et al 2014^(8)^ | * | * | * |  | * | * | * | * | * | 8 |
| Bates et al 2018^(10)^ | * | * | * |  |  | * | * | * | * | 7 |
| Tseng et al 2011^(9)^ | * | * | * |  |  | * | * | * | * | 7 |
| Zhou et al 2010^(13)^ | * | * | * |  |  | * | * | * | * | 7 |
| Datta et al 2017^(11)^ | * | * | * |  | * | * | * | * | * | 8 |
| Lepechoux et al 2013^(12)^ | * | * | * |  | * | * | * | * | * | 8 |
| Gronchi et al 2009^† (16)^ | * | * | * |  |  | * | * | * | * | 7 |
| Gronchi et al 2009^‡ (16)^ | * | * | * |  |  | * | * | * | * | 7 |
| Gronchi et al 2012^† (17)^ | * | * | * |  |  | * | * | * | * | 7 |
| Gronchi et al 2012^‡ (17)^ | * | * | * |  |  | * | * | * | * | 7 |
| Stahl et al 2017^† (15)^ | * | * | * |  | * | * | * | * | * | 8 |
| Stahl et al 2017^‡ (15)^ | * | * | * |  | * | * | * | * | * | 8 |
| Klooster et al 2016^† (20)^ | * | * | * |  |  | * | * | * | * | 7 |
| Klooster et al 2016^‡ (20)^ | * | * | * |  |  | * | * | * | * | 7 |
| Nathan et al 2009^(5)^ | * | * | * |  |  | * | * | * | * | 7 |
| Berger et al 2018^(22)^ | * | * | * |  |  | * | * | * | * | 7 |
| Chouliaras et al 2019^a (21)^ | * | * | * |  | * | * | * | * | * | 8 |
| Miura et al 2015^a† (18)^ | * | * | * |  | * | * | * | * | * | 8 |
| Miura et al 2015^a‡ (18)^ | * | * | * |  | * | * | * | * | * | 8 |

**Notes:** ^a^ Propensity score matched (PSM); ^†,‡^ Adjuvant radiotherapy (†) and adjuvant chemotherapy (‡) in the same study

**Table S2. Begg’s and Egger’s Test for Publication Bias**

| **Categories** | **Begg’s Test** | | **Egger’s Test** | |
| --- | --- | --- | --- | --- |
|  | **Z-value** | **Pr>/z/** | **T-value** | **Pr>/t/** |
| **OS** | | | | |
| Adjuvant radiotherapy | 0.44 | 0.661 | -1.29 | 0.221 |
| Adjuvant chemotherapy | 0.00 | 1.000 | -0.06 | 0.953 |
| **RFS** | | | | |
| Adjuvant radiotherapy | 1.02 | 0.308 | -2.30 | 0.148 |
